# Prevention of therapy-related malignances in cancer survivors

**DOI:** 10.18632/oncotarget.26781

**Published:** 2019-03-15

**Authors:** Gennady A. Belitsky, Kirill I. Kirsanov, Ekaterina A. Lesovaya, Marianna G. Yakubovskaya

**Affiliations:** Blokhin National Medical Research Center of Oncology, Moscow, Russia

**Keywords:** cancer, therapy-related malignances, chemo-prevention, plant polyphenols

Genotoxic cytostatics currently used in anti-cancer therapies are carcinogenic. These agents cause appearance of new chemotherapy-resistant primary tumors in a cohort of previously cured patients. Given that advances in early diagnosis and therapy allow more than 60% of cancer patients to live for five or more years, there are millions of people worldwide are at some degree of carcinogenic risk [1]. Unlike sporadic carcinogenesis, the characteristics of the iatrogenic one leading to the appearance of secondary malignances are known. They include the origin of carcinogenic drugs, the time of their action and the approximate duration of the latent period. In terms of traditional two-stage chemical carcinogenesis model, the cytotoxic treatment corresponds to the initiation step, which is followed by the latent period (i.e. the promotion step), during which cell clones undergo varying degrees of malignant transformation. Since therapy-induced tumors occur in a small group of survivors, prior to the beginning of cytostatic regimens, there is a great need for identification and stratification of patients who are pre-disposed to the therapy-induced carcinogenesis. Two groups of such patients could be characterized by genetic peculiarities in either drug-metabolizing or DNA repair enzymes. The first group represents individuals with predominance of enzymes activating cytostatic agents over enzymes responsible for detoxification. Activating enzymes include the cytochrome P450 isoforms CYP 2B6, 3A4, 3A5, 2C9 and cytochrome C reductase important for Cyclophosphamide activation, as well as carboxylesterases CES1 and CE - for Irinotecan. Etoposide is mainly activated by CYP3A4 and myeloperoxidase, whereas metabolic fates of Doxorubicin and Daunorubicin is defined by CYP3A5, NADH cytochrome P450 reductase, NADH dehydrogenase and xanthinoxidase. Tamoxifen genotoxic effect depends on CYP2D6 and CYP3A4 activity. The inactivating enzymes for all these cytostatics are represented by glutathione-S-transferases (including GSTα, GSTμ, GSTθ and GSTπ), NADPH: quinone reductases, UDP-glucuronyl transferases, aldoketoreductases, superoxide dismutase and other enzymes.

The second group of patients with increased risk of drug-induced carcinogenesis is characterized by insufficient activity of enzymes responsible for DNA repair, in particular, BRCA2 (FANCD1), BRIP1(FANCJ), PALB2(FANCN), RAD51C (FANCO), ERCC4(FANCQ), and others. This cohort of patients requires special attention since high sensitivity to the toxic effects of cytostatics of standard doses for them is excessive in terms of both treatment and carcinogenic risk. For example, in cells of patients with Fanconi anemia, the same dose of cyclophosphamide gives 10 times more N7-guanine adducts responsible for cytotoxic and carcinogenic effects than in patients without this syndrome [2].

The use of protective drugs for the prevention of secondary carcinogenesis during chemotherapy has not resulted in much success, since selective protection of normal cells turned out to be as difficult as development of the drugs with selective genotoxic effect on the tumor cells. In this regard, the main efforts in prevention of drug-induced carcinogenesis is focused on interventions during the latent period, when some of the precursors of tumor cells are at different stages of malignant transformation. Taking into consideration that adducts of some cytotoxic drugs such as platinum derivatives can be detected in the body for up to 10 years [3]. During the latent period, it is of utmost importance to prevent the occurrence of additional mutations that would provide additional hits for completion of malignant genotype. Furthermore, it is also important to inhibit proliferation and progression of already transformed clones. Means to prevent additional mutagenesis during this latent period mainly reiterate the recommendations for primary cancer prevention. They include the elimination of occupational hazards and harmful habits, especially smoking.

Recent interest in effective chemoprevention against iatrogenic carcinogenesis has emerged. These chemopreventive approaches may include the use of natural anti-carcinogens, in particular, plant polyphenols, which can simultaneously affect diverse steps of signal transduction, gene transcription and translation that underlie carcinogenesis. Plant polyphenols modulate cell signal pathways in such a way that not only inhibits mutagenesis, but also inactivate the epigenetic processes, which drive proliferation and survival of transformed cells [4, 5]. Plant polyphenols are known to inhibit free radical reactions. Among these strong antioxidants are tea catechins and quercetin that activate NRF2 transcription factor. In turn, NRF2 is associated with cytoprotectors that stimulate the biosynthesis of antioxidants - hemoxygenase 1, NAD (P) H-quinone oxidoreductase, glutathione transferase and other enzymes that inactivate oxidative radicals. As a result, the resistance of cells to xenobiotics, which provoke additional mutagenesis in premalignant cells, significantly increases [6]. Plant polyphenols also inhibit inflammation. Green tea catechins, indole-3-carbinol and sulfarofan have the most pronounced inhibitory effect on inflammation, affecting the AhR receptor, which in turn inhibits NF-κB by IRAK kinase supression, as well as direct inhibiting of interleukins 6,8, TNF -α and cyclooxygenase-2 [7]. Furthermore, plant polyphenols were shown to stimulate apoptosis. Studies in cell cultures and *in vivo* demonstrated the ability of green tea catechins to induce apoptosis of tumor cells associated with ubiquitin-dependent degradation of cyclin D1 and simultaneous activation of the p21 transcription [8]. Food flavonoids and isocyanates from cruciferous vegetables inhibit cell proliferation and suppress pathological hyperplasia induced by a multitude of unrelated stimuli. Interruption of proliferative signals occurs along many signaling pathways and, primarily, through the AhR receptor. Normally, ligand-unbound AhR stimulates cell cycle progression by forming a complex with CDK4/CCND1 kinase, subsequent hyperphosphorylation of RB1 and release of the transcription factor E2F. As a result of AhR binding to the flavonoid, the receptor conformation changes, it dissociates from the complex with CDK4/CCND1 and binds to hypomethylated RB1. This new complex prevents cells from entering the S phase by inhibiting the expression of EP300 proteins and E2F1-dependent genes CDK2 and CCNE. In addition, AhR bound to the ligand forms a complex with ARNT and stimulates transcription of the cell cycle inhibitor CDKNB1 [9]. Moreover, flavonoids were shown to affect cytoskeleton and malignant cell motility and invasion. As malignant cells express metalloproteinases to disrupt the basement membrane for invasion, studies in tissue culture and in transplanted tumors showed that this process can be inhibited by green tea plant polyphenols. It is likely that this effect contributes to prevention of growth and spread of malignant clones [10, 11]. Furthermore, plant polyphenols inhibit neoangiogenesis through variety of mechanisms. One of these mechanisms related to blockage of VEGF and its receptors (VEGFB, VEGF-C and VEGF-D) was demonstrated for gallate epigallocatechin that binds the transcription factor AP-1 and subsequently inactivates VEGF. The distal proteins of this cascade - VE-cadherin and Akt - are also targets of green tea catechins [12]. The ability of flavonoids to selectively inhibit transformed cells was noted in many studies that focused on particular characteristics of malignant cells. One of the explanations for this selectivity is that the initially steps of cell transformation can be limited by changes in a few systems, for example, specific oncogene activation, suppressor inhibition, or related epigenetic changes. Disruption of these specific signaling pathways should not significantly affect the proliferation of normal cells. Molecular mechanisms of plant polyphenols effects require additional studies. The use of these compounds are promising as a part of a comprehensive strategy for the prevention of cytostatics-induced malignancies.
